# Protocol for a systematic review of interventions addressing health literacy to improve asthma self-management

**DOI:** 10.1038/s41533-019-0125-y

**Published:** 2019-05-08

**Authors:** Hani Salim, Ingrid Young, Sazlina Shariff Ghazali, Ping Yein Lee, Siti Nurkamilla Ramdzan, Hilary Pinnock

**Affiliations:** 1 NIHR Global Health Research Unit on Respiratory Health (RESPIRE), Usher Institute of Population Health Sciences and Informatics, The University of Edinburgh, Edinburgh, UK; 2 Department of Family Medicine, Faculty of Medicine and Health Sciences, Universiti Putra Malaysia, Serdang, Malaysia; 3 Centre for Biomedicine, Self and Society, Usher Institute of Population Health Sciences and Informatics, University of Edinburgh, Edinburgh, UK; 4 Department of Primary Care Medicine, Faculty of Medicine, University of Malaya, Kuala Lumpur, Malaysia

**Keywords:** Asthma, Outcomes research

## Introduction

Supported self-management for asthma (written action plans and regular review) is highly effective at improving control and reducing acute attacks;^1–3^ however, globally this is challenging to implement both in adults and children.^4–7^ One particular challenge is the need to tailor support for people with limited health literacy.

Limited health literacy is a universal problem, especially (but not only) in low-and-middle-income countries (LMICs).^8–10^ For example, nearly 90% of adults with type 2 diabetes attending primary care clinics in Malaysia^11^ and a third of teachers in Sri Lanka^12^ were assessed as having limited health literacy compared to about half the general population in a European survey.^9^ Use of different assessment techniques and sampling of different populations make it difficult to compare these results, though it is clear that this is a problem in all countries.

Derived from a systematic content analyses of 19 papers, Sorensen et al.^13^ describes health literacy as people’s knowledge, motivation and competence to assess, understand, appraise and apply health information (see Table 1 for definitions). These processes are vital to enable people to make considered judgments and decisions about healthcare and health promotion, which can improve quality of life and health outcomes such as reducing morbidity.^13,14^

**Table 1:** Definition of terms

Terms	Definition
Self-management^34^	The tasks that individuals must undertake to live with one or more chronic conditions. These tasks include having the confidence to deal with medical management, role management and emotional management of their conditions
Health literacy^13^	Health literacy is linked to literacy and entails people’s knowledge, motivation and competences to access, understand, appraise and apply health information in order to make judgments and take decisions in everyday life concerning healthcare, disease prevention and health promotion to maintain or improve quality of life during the life course
Severe asthma attacks^28^	Events that require urgent action on the part of the patient and physician (e.g. a course of oral steroids) to prevent a serious outcome, such as hospitalisation or death from asthma

Health literacy is linked to functional literacy skills such as the ability to read and count.^10^ Among people with asthma for example, difficulty in reading is associated with improper use of inhalers and poor disease knowledge.^15^ Poor numeracy skills are associated with increased emergency visits and hospitalisations among people with asthma.^16^ Studies have also associated limited health literacy with erroneous health beliefs and poor adherence to self-management activities.^17,18^ Without health literacy skills, self-management will be difficult.^3,19^ A review of causal pathways suggests that health literacy is not related to health outcomes in a linear function. Health literacy influenced three aspects of healthcare: (i) access and utilisation of healthcare, (ii) patient–provider interactions, and (iii) self-care.^14^ As an example, worsening of symptoms despite initial self-management will motivate people with asthma to seek medical care. However, in people with limited health literacy, poor knowledge of an impending exacerbation may delay early medical attention and result in poor health outcomes.

There are two previous systematic reviews looking at interventions for adults and children/carer with limited health literacy. Sheridan et al. included three asthma-related interventions, two of which reduced emergencies and hospital admissions^20^ but none were randomised control trials. Schaffler et al. included one asthma-related study in their review of self-management interventions among people from low socioeconomic groups (fewer than half were in populations with poor health literacy).^21^ Both reviews conclude that mixed-strategy interventions targeting three to four self-management skills (specifically including problem-solving) are more likely to be effective than single component self-management interventions for people with limited health literacy. These reviews have limitations: the searches in Sheridan et al. were completed in 2011, and half the populations included in the more recent review by Schaffler et al. were defined by low income (as opposed to limited health literacy).^20,21^

To inform the development of supported self-management for asthma in LMICs, we aimed to systematically search and synthesise the evidence for asthma self-management interventions targeted at people with limited health literacy, in order to assess their clinical effectiveness and to identify the behaviour change strategies that are associated with effective programmes.

## Methods

We will follow the procedures described in the Cochrane Handbook for Systematic Reviews of Interventions.^22^ The PRISMA-P (Preferred Reporting Items for Systematic reviews and Meta-Analyses for Protocols) checklist has been used as a framework for this protocol.^23^ The review is registered with the International Prospective Register of Systematic Review (PROSPERO): CRD 42018118974.

### Search strategy

We will search 10 electronic databases (listed in Table 2). The search strategy uses medical subject headings (MeSH) and text words related to health literacy, asthma, self-management and controlled trial (Supplementary Table 1). The search will commence from 1990 onwards. Although the term “health literacy” was introduced in 1970,^24^ the concept of asthma self-management was first recommended in the asthma guidelines in 1990.^25^

**Table 2:** PICOS descriptions and definitions

PICOS	Descriptions and definitions
Participants/population	Participants will be those with physician-diagnosed asthma (children, adolescents, adults and the elderly) or their parents/carers.
Intervention(s), exposure(s)	Asthma self-management interventions targeted at participants with limited health literacy level will be included. We will note how the authors define limited health literacy and included those interventions that meet our definition (see Table 1). We will also include interventions that include training of healthcare practitioners to give them skills to teach self-management to people with limited health literacy, if the outcomes include the impact on the patient.
Comparator	Typically, the comparator will be usual care, but may also be alternative self-management strategies, e.g. self-management interventions not targeting health literacy.
Outcomes	For **primary outcomes**, we are interested in both health and implementation outcomes.^35^ As recommended by the European Respiratory Society/American Thoracic Society (ERS/ATS) Task Force report on asthma outcomes,^28^ the primary health outcomes include the following: ∘ Current asthma control (e.g. using a validated questionnaire such as the Asthma Control Questionnaire^36^ or Asthma Control Test^37^) ∘ Future risk (e.g. the number of severe attacks, steroid courses, emergency department visits or hospitalisations). We will use the ERS/ATS definition of ‘severe asthma exacerbations’ (see Table 1): The primary implementation outcomes will be the following: adoption of the interventions (e.g. proportion of participants taking up the intervention, provided with an action plan) and adherence to interventions (e.g. frequency of usage of an action plan). For **secondary outcomes**, we will include practical self-management measures such as self-efficacy, activation, empowerment, correct inhaler use, improvement in knowledge, health literacy outcomes and impact indicators of interventions, such as cost-effectiveness, fidelity and sustainability.
Setting	We will include any clinical or community-based setting. These settings can be based in developed or developing nations (specifically including LMICs).
Study design	We will include controlled experimental studies: randomised controlled trials (RCTs), controlled clinical trials, controlled before-and-after studies and interrupted time-series designs.
Database searched	1. MEDLINE: Medical Literature 2. EMBASE: Excerpta Medica dataBASE 3. CINAHL Plus: Cumulative Index to Nursing and Allied Health Literature 4. PsycINFO: Database of abstracts of literature in the field of psychology 5. AMED: Allied and Complementary Medicine Database 6. BNI: British Nursing Index 7. Cochrane Library: Database of Abstracts of Reviews of Effects, Cochrane Database of Systematic Reviews (CDSR) and Cochrane Central Register of Controlled Trials (CENTRAL) 8. Web of Science Core Collection (including International Scientific Indexing (ISI) Proceedings) 9. ScienceDirect 10. Global Health

Reference lists will be examined for other relevant studies, and we will undertake forward citation of any included studies. For unpublished and in-progress studies, we will search the WHO ICTRP (World Health Organisation International Clinical Trials Registry Platform: https://www.who.int/ictrp, and US National Institutes of Health Ongoing Trials Register ClinicalTrials.gov: https://www.clinicaltrials.gov) for relevant trials and will contact the author if a publication was not found. We will note any conference abstracts and look for subsequent publications. We will contact experts in the area to enquire about related trials. We do not plan manual searches, unless a journal emerged from the electronic searches as focusing specifically in this area. There will be no language restrictions; where possible, we will arrange translation.

### Eligibility criteria

We will search the databases using the PICOS criteria and the operational definitions (Tables 2 and 3). We were aware that interventions may not use our chosen terminology, so will use the definitions in Table 1 to confirm eligibility.

**Table 3:** Operational definitions

Terms	Definition
People with limited health literacy	We defined intervention as one that included people with limited health literacy quantitatively and using evidence-based approaches. Quantitatively, study populations that measured health literacy level using validated tools and included ≥40% of people with limited health literacy in the trial are included. We will include study populations that include individuals with high risk of limited health literacy through publications of evidence in systematic review reports and qualitative studies. This will include the following: 1) Immigrants 2) Ethnic minorities 3) Illiterates
Types of interventions (1) Self-management	We will include any asthma-self management interventions within the taxonomy of the self-management support components suggested by Taylor et al.^3^. a) Direct components (delivered directly to patients and/or carers) such as education, action plans and practical support with adherence. b) Indirect components: healthcare or social care at a professional level (delivered to individual healthcare professionals or social care professionals) such as equipment, feedback and review. c) Indirect components: delivered at an organisational level such as prompts using paper or electronic reminders.
(2) Addressing health literacy	We will included any interventional designs that are aimed at improving health literacy, as suggested by Sheridan et al.^20^: a) Presenting written information differently (e.g. essential information first) b) Presenting numerical information differently (e.g. the highest number is better) c) Using icons, symbols and graphs d) Presenting information pitched at a lower literacy level (e.g. primary school comprehension) e) Use of videos f) Literacy training for patients and physicians g) Implementing comprehension skills to enable self-care

### Selection process

The literature search results retrieved from the electronic databases will be uploaded to DistillerSR Software to enable collaboration between reviewers. This software facilitates screening, de-duplication and overall management of the search results. In an initial training process, the two reviewers (H.S. and S.N.R.) will screen a sample of 100 titles and abstracts, compare the results and, in discussion with the study team, refine the inclusion and exclusion criteria. Title and abstract screening will then be undertaken independently by the two reviewers.

We will obtain the full text of potentially relevant studies, and both reviewers (H.S. and S.N.R.) will independently assess them for eligibility. We will identify and record the reasons for exclusion of ineligible studies. Disagreements or uncertainties at any stage will be resolved by discussion within the team (H.P., I.Y., S.G.S. or P.Y.L.).

Studies that have multiple publications (e.g. protocol, trial findings, process evaluations, qualitative studies, translations) will be treated as one study; however, reference will be made to the different publications. The process of selection will be summarised using a PRISMA flow diagram to ensure transparency.^23^

### Data extraction and management

To ensure the quality of description of interventions, we will use the Effective Practice and Organisation of Care (EPOC) group recommendations for describing interventions^26^ and the Template for Intervention Description and Replication (TIDieR) checklist.^27^ Data will be extracted independently by H.S. and S.N.R. into a piloted data extraction form and presented under the following headings:

- Methods: study design, number of study centres and location, study setting, withdrawals, date of study, follow-up.
- Participants: number, age, gender, severity of condition, inclusion criteria, exclusion criteria, other relevant characteristics.
- Outcomes: Reflecting guideline recommendations,^19,28^ our primary health outcomes were current symptom control and risk of acute attacks. Our primary implementation outcomes are measures of adoption of the intervention. Definitions, methods of assessment and secondary outcomes are in shown Table 2.
- Components of the intervention. We will use the behaviour change model to categorise the components of the intervention enabling us to identify strategies associated with effective programmes.^29^

To ensure that the tables are interpreted consistently and all relevant data are captured, the two reviewers (H.S. and S.N.R.) will complete the data extraction process and a third reviewer arbitrated, if necessary. We will contact the authors if sufficient information was not found within the included paper(s).

### Assessment of risk of bias in the included studies

All included studies will be assessed independently for potential risk of bias by the two reviewers (H.S. and S.N.R.). We will use the Cochrane Risk of Bias tool,^22^ and the guidance from the EPOC group,^26^ to assess selection, performance, detection, attrition, reporting and other potential sources of bias.^22^ The risk of bias for each domain will be classified as ‘low’, ‘high’ or ‘unclear’ based on the information available.^22^ We will generate ‘risk of bias’ summary graphs and figures using Review Manager (RevMan 2012).^30^

### Assessment of risk of the quality of evidence

We will use the Grading of Recommendations Assessment Development and Evaluation (GRADE) approach to assess the quality of evidence related to each primary outcome.^31^ We will assess the overall quality of evidence for each outcome using five factors: risk of bias, inconsistencies, indirectness, imprecision of effect estimates and publication bias.^31^ We will downgrade the evidence from ‘high quality’ by one level for serious and by two level for very serious outcomes (high, moderate, low, very low).^31^

### Data analysis

Our data analysis will address the two aspects of our aim (effectiveness and identification of strategies associated with effective programmes).

#### Analysis of effectiveness of the intervention

We will examine our data for the primary health and implementation outcomes to assess their suitability for meta-analysis. For dichotomous outcomes we will use risk ratio (RR), and for continuous outcomes, we will use mean difference (MD), standardised mean difference (SMD) or mean change difference (MCD). All effect estimates will be expressed using 95% confidence intervals (CI). We will pool data using a random-effects model with the Review Manager 5 (RevMan 5)^31^ software, testing for heterogeneity with Cochran’s *Q* and *I*^2^ statistics, and publication bias using Begg’s and Egger’s tests to assess funnel plot asymmetry.^32^ Our scoping work, however, suggested that studies will vary substantially in design, target populations, outcomes measured and duration of follow-up, so that meta-analysis may not be appropriate. If this is the case, we will undertake a narrative synthesis, potentially illustrating the findings for key outcomes with a Harvest plot.^33^

#### Identification of strategies associated with effective programmes

The second aim will seek to identify the strategies, as described in the Behaviour Change Wheel model,^29^ that are associated with effectiveness. The core of this model involves strategies that influence capability, opportunity and motivation, and that result in behaviour change (COM-B).^29^ Capability is defined as the individual’s psychological and physical capacity to enact a behaviour.^29^ It includes possessing the required knowledge and skills. Motivation is defined as those processes that contribute towards both reflective and automatic mechanisms that activate or inhibit behaviour.^29^ Opportunity is defined as aspects of the physical and social environment that lie outside the individual and that prompt or make the behaviour possible.^29^

Using the nine intervention functions of the Behaviour Change Wheel, we will map components of the interventions in the included studies to the outcomes. This will enable us to identify components common to successful interventions, to understand the potential mechanism of action of the interventions, and also to identify gaps around three essential conditions that govern behaviour system—capability, opportunity and motivation.^29^

### Dissemination

We will submit the findings of this study to peer-reviewed journals and conferences for presentations. Other methods of dissemination will included innovative dissemination channels of RESPIRE (websites and Twitter) to raise awareness of our publications. Apart from formal routes of dissemination, as researchers from various backgrounds, we will use our professional networks, stakeholder engagement activities, and patient and public involvement channels to raise awareness of the work we have done in this area.

## Discussion

Limited health literacy is associated with erroneous health beliefs, inadequate inhaler technique, limited adherence to self-management activities and sub-optimal clinical outcomes.^17,18^ Although the relationship of health literacy and supported asthma self-management is likely to be complex, interventions that address literacy needs may improve asthma outcomes in this vulnerable population. The findings of the review will inform the development of self-management support interventions targeting people with asthma who have limited health literacy.

## Supplementary information

Supplemantary 1: Search terms

## Data Availability

Data sharing is not applicable. This article is a protocol without any dataset generated or analysed.

## Supplementary information

**Supplementary information** accompanies the paper on the *npj Primary Care Respiratory Medicine* website (10.1038/s41533-019-0125-y).
