# Supplementary material for: Protocol for a systematic review of interventions addressing health literacy to improve asthma self-management
Source: NPJ Prim Care Respir Med. 2019 May 8;29:18. doi: 10.1038/s41533-019-0125-y (PMC6506542; doi:10.1038/s41533-019-0125-y)
Supplement: Supplementary file 1 — Supplemantary 1: Search terms [file 41533_2019_125_MOESM1_ESM.docx]

| SUPPLEMENTARY 1:SEARCH TERMS | | | | |
| --- | --- | --- | --- | --- |
| Database | Terms used | | | |
|  | Health literacy | Asthma | Self-management | Study design |
| MEDLINE  AMED  PSYINFO  EMBASE  GLOBAL HEALTH | 1. health litera$2.af. 2. medical literacy.af. 3. (health and literacy).ti. 4. (functional and health and literacy).tw. 5. low-litera$2.ti. 6. litera$2.ti. 7. illitera$2.ti. 8. reading/ 9. comprehension/ 10. motivation/ 11. health promotion/ 12. health education/ 13. patient education/ 14. audiovisual aids/ 15. communication barriers/ 16. communication/ 17. social skills/ 18. health knowledge,attitudes,practice/ 19. attitude to health/ 20. comprehension/ and educational status/ 21. (family and literacy).ti. 22. (drug labeling.tw. or prescriptions, drug/) and comprehension.tw. 23. ((asthma) and (literacy or comprehension)).ti. 24. (adult and (educational status or (educational and status) or literacy)).tw. 25. (limited and (educational status or (educational and status) or literacy)).tw. 26. (patient$1 and (educational status or (educational and status) or literacy)).tw. 27. (patient$1 and (comprehension or understanding)).ti. 28. Or/1-27 | 1. Exp Asthma/ 2. Bronchial Spasm/ 3. Bronchoconstriction/ 4. ((bronchial* or respiratory or airway* or lung*) ADJ3 (hypersensitive* or hyperreactiv* or allerg* or insufficiency)) 5. Bronch* ADJ3 (constrict* or spas*) 6. Asthma.tw. 7. Wheeze.tw. 8. Bronchoconstrict.tw. 9. Antiasthma.tw. 10. Anti-asthma.tw. 11. Respiratory sounds/ 12. Bronchial hyperreactivity/ 13. or/1-12 | 1. Exp Self care/ 2. Exp Communication/ 3. Exp Professional Family Relations/ 4. Exp Telephone/ 5. Exp Professional Patient Relations/ 6. Exp Health education/ 7. Exp Attitude of health personnel/ 8. Exp Cellular phone/ 9. Exp Patient education as topic/ 10. Exp Handheld computer/ 11. Exp Self efficacy/ 12. Exp Activities of Daily Living/ 13. Exp Self help devices/ 14. Exp Community health services/ 15. Exp Rehabilitation/ 16. (Self ADJ2 (car* or manag* or help or administ* or monitor* or medicat*)) or self-car* or self-manag* or self-help or self-administ* or self-monitor* or self-medicat* or selfcar* or selfmanagement or selfhelp or selfadminist* or selfmonitor* or selfmedicat* 17. SM or SMS 18. Responsib* or Autonom* 19. Manag* or copes or coping 20. (Disease management) 21. (expert patient) 22. (professional or clinician) ADJ2 development 23. Educat* or training or skill* or knowledge 24. Confidence or self-efficacy 25. (Access* or provi*) ADJ3 (information or records or results) 26. Monitor* or self-monitor* or selfmonitor* 27. ((patient or individual* or person* or client*) ADJ3 (remind* or feedback)) 28. (Tele ADJ2 (health or medicine or care)) or tele-health or tele-medicine or tele-care or telehealth or telemedicine or telecare 29. ((Short message service) or SMS or (mobile phone) or (text message*)) 30. (home or environment* or living or assistive) ADJ2 (adaptation or modif* or equipment or technolog*) 31. (Care plan*) 32. (Action plan*) 33. Hypno* ADJ1 (self or home) 34. (cognitive or psychological or interpersonal or relaxation or biofeedback) ADJ3 (therap* or intervention* or program*) 35. CBT 36. Psychoeducation* 37. (Peer or patient or emotional or social or psychosocial) ADJ1 (support or group) 38. (Expert patient) 39. Financial ADJ1 control 40. (personal health budget*) 41. (Financial or monetary or payment* or discount or service*) ADJ5 incentiv* 42. Or/1-42 | 1. randomized controlled trials as topic/ 2. randomized controlled trial/ 3. random allocation/ 4. double blind method/ 5. single blind method/ 6. clinical trial/ 7. clinical trial, phase i.pt 8. clinical trial, phase ii.pt 9. clinical trial, phase iii.pt 10. clinical trial, phase iv.pt 11. controlled clinical trial.pt 12. randomized controlled trial.pt 13. multicenter study.pt 14. (quasiexperimental or quasi experimental or pseudo experimental or controlled before-and-after or interrupted time series design*).tw. 15. exp clinical trials as topic/ 16. (clinical adj trial$).tw 17. ((singl$ or doubl$ or treb$ or tripl$) adj (blind$3 or mask$3)).tw 18. (control group).tw 19. (usual care).tw 20. randomly allocated.tw 21. (allocated adj2 random$).tw 22. or/1-21 |
| CINAHL | S1 health literacy  S2 AB medical literacy  S3 AB health AND AB literacy  S4 AB functional AND AB health AND AB literacy  S5 TI low literacy  S6 TI literacy  S7 TI illiteracy  S8 TI illiterate  S9 AB reading  S10 AB comprehension  S11 AB motivation  S12 AB health promotion  S13 AB health education  S14 AB patient education  S15 TX audiovisual  S16 TX audio visual  S17 AB communication barrier  S18 AB communication  S19 AB communication skills  S20 AB social skills  S21 TX health knowledge, attitudes, practice  S22 AB attitude to health  S23 AB comprehension AND AB educational status  S24 TI family AND TI literacy  S25 AB ( adults or adult ) AND AB (educational status or literacy)  S26 AB ( drug labeling or prescription or drug ) AND AB comprehension  S27 TI asthma AND TI (literacy or comprehension)  S28 AB limited AND (educational status or literacy )  S29 AB ( patients or clients) AND AB ( educational status or literacy)  S30 TI ( patients or clients ) AND TI  (comprehension or understanding )  S31 OR/S1-S30 Limiters - Publication Year: 1990-2018; Human Search modes – Find all my search terms | S1 AB asthma  S2 AB bronchial asthma  S3 AB bronchial spasm  S4 AB bronchoconstriction  S5 AB bronchial or respiratory or airway or lung or hypersensitive or hyperactive or insufficiency  S6 AB wheezes and asthma  S7 AB respiratory sound  S8 OR/S1-S7 Limiters – Publication Year: 1990-2018; Human Search modes – Find all my search terms | S1 AB self care  S2 AB professional family relations  S3 TX telephone  S4 AB professional family relations  S5 AB attitude of health personnel  S6 AB cellular phones or cellphones or smart phones  S7 AB handheld devices OR AB handheld technology OR AB handheld devices in healthcare  S8 AB self-efficacy  S9 AB activities of daily living or ADL  S10 AB self-help devices  S11 AB community health services  S12 TI rehabilitation or therapy or treatment  S13 AB responsibility OR AB autonomy  S14 AB (manage or management) OR AB (cope or coping or coping strategies or coping mechanisms or coping skills)  S15 AB disease management  S16 AB expert patient OR AB expert patient programme OR AB expert patient program  S17 AB (professional or clinician) AND AB development  S18 AB education OR AB training OR AB (skill or skills) OR AB knowledge  S19 AB confidence OR AB self-efficacy  S20 AB (access or provide) AND AU (information or records or results)  S21 AB monitor OR AB self monitoring OR AB self-monitoring behaviour  S22 AB (patient or individual or person or client) AND AB (remind or feedback)  S23 AB telehealth or telemedicine or telemonitoring or telepractice or telenursing or telecare  S24 AB short message service OR AB (sms or text messaging) OR AB (mobile phone or cell phone or smartphone or mobile technology)  S25 AB ( home or environment or living or assistive ) OR AB (adaptation or modifications or equipment or technology)  S26 AB care plan  S27 AB action plan  S28 AB (cognitive or psychological or interpersonal or relaxation or biofeedback ) AND AB ( therapy or treatment or intervention or program)  S29 AB cbt or cognitive behavioral therapy or cognitive behaviour therapy  S30 TI psychoeducational intervention or psycho education or psycho-education  S31 AB (peer or patient or emotional or social or psychosocial) AND AB (support or guidance or help or assistance)  S32 AB financial AND AB control  S33 AB personal health budgets  S34 AB (financial or monetary or payment or discount or service ) AND AB (incentives or rewards or motivation)  S35 OR/S1-S34 Limiters – Publication Year: 1990-2018; Human Search modes – Find all my search terms | S1 TI randomized controlled trials or RCT or randomised control trials  S2 AB randomized controlled trials or RCT or randomised control trials  S3 TX Random allocation  S4 TX Double blin method  S5 TX Single blind method  S6 AB clinical trials, randomized  S7 AB controlled clinical trial  S8 AB controlled clinical trial [pt]  S9 AB randomized controlled trial [pt]  S10 AB multicenter study [pt]  S11 AB clinical trials [pt]  S12 TI clinical trials  S13 TX control group OR TX usual care  S14 TX random allocation OR TX randomly assigned  S15 TX phase 1 clinical trials OR TX phase 2 study OR TX phase 3 clinical trials OR TX phase 4  S16 AB quasiexperimental or Ab quasi experimental or AB pseudo experimental or AB controlled before-and-after or AB interrupted time series design  S17 OR/S1-S16 |
| BNI | S1 noft(asthma) OR noft(asthma bronchial) | S1 noft((health AND litera*) OR (litera*) OR (health litera*) OR (medical litera*) OR (health litera*) OR (functional AND health AND litera*) OR (low litera*) OR (illitera*) OR (reading OR read) OR (comprehension) OR (motivation) OR (health promotion) OR (health education) OR (communication barrier*) OR (audiovisual aid*) OR (communication*) OR (social skill*) OR (health knowledge AND attitude AND practice) OR (attitude* to health) OR (comprehension AND education* status) OR (family AND litera*)) OR noft((drug labelling OR prescription*) AND (comprehension)) OR noft((asthma) AND (litera* OR comprehension)) OR noft((adult) AND (education* status OR litera*)) OR noft((limited OR poor) AND (education* status OR literacy)) OR noft((patient*) AND (education* status OR literacy)) OR noft((patient*) AND (comprehension OR understanding)) | S1 noft((Self care OR self-care) OR (professional family relation*) OR (telephone) OR (professional patient relation*) OR (attitude* of health personnel*) OR (cellular phone) OR (handheld computer) OR (self efficacy) OR (self efficacy OR self-efficacy OR confidence) OR (ADL) OR (activit* of daily living)) OR noft((self-help devices) OR (community health service*) OR (rehabilitation) OR (responsib* OR autonomy*) OR (manag* OR cope* OR coping) OR (disease management) OR (expert patient) OR (educat* OR training OR skills* OR knowledge)) OR noft((monitor* OR self-monitor* OR selfmonitor*) OR (tele-health OR tele medicine OR tele-care OR telehealth OR telemedicine OR telecare) OR (short message service* OR SMS OR mobile phone* OR text message*) OR audiovisual* OR (care plan OR action plan) OR (personalised action plan) OR (CBT) OR (psychoeducation*) OR (personal health budget*) OR (financial AND control)) OR noft((professional* OR clinician*) AND (development)) OR noft((patient* OR individual* OR person* OR client*) AND (remind* OR feedback)) OR noft((access* OR provi*) AND (information OR record* OR result*)) OR noft((home OR environment* OR living OR assistive) AND (adaptation OR modif* OR equipment OR technolog*)) OR noft((cognitive OR psychological OR interpersonal OR relaxation OR biofeedback) AND (therap* OR intervention* OR program*)) OR noft((peer OR patient OR emotional OR social OR psychosocial) AND (support OR group*)) OR noft((financial OR monetary OR payment* OR discount OR service*) AND (incentiv*)) | S1 noft (randomized controlled trial*) OR (controlled clinical trial*) OR (quasiexperimental) OR (quasi experimental) OR (pseudo experimental) OR (controlled before-and-after) OR (interrupted time series design) |
| WEB OF SCIENCE | 1. TS=(health AND litera*) OR TS=(litera*) OR TI=(health litera*) OR TS=(medical litera*) OR SU=(health litera*) OR TS=(functional AND health AND litera*) OR TI=(low litera*) OR TI=(illitera*) OR TS=(reading OR read) OR TS=(comprehension) OR TS=(motivation) OR TS=(motivation) OR TS= (health promotion) OR TS=(health education) OR TS=(communication barrier*) OR TS=(audiovisual aid*) OR TS=(communication*) OR TS= (social skill*) OR TS=(health knowledge AND attitude AND practice) OR TS=(attitude* to health) OR TS=(comprehension AND education* status) OR TI=(family AND litera*) 2. TS=(drug labelling OR prescription*) AND TS=(comprehension) 3. TS=(adult) AND TS=(education* status OR litera*) 4. TS=(limited OR poor) AND TS=(education* status OR literacy) 5. TS=(patient*) AND TS=(education* status OR literacy) 6. TI=(patient*) AND TI=(comprehension or understanding) 7. OR/1-6 | 1. TS=(asthma) OR TS=(bronchial spasm) OR TS=(bronchoconstriction) OR TS=(wheez*) OR TS=(bronchoconstrict) OR TS=(antiasthma) OR TS= (anti-asthma) OR TS=(respiratory sound*) OR TS=(bronchial hyperreactiv*) 2. TS=(bronchial* OR respiratory OR airway* OR lung*) AND TS=(hypersensitive* OR hyperreactiv* OR allerg* OR insufficien*) 3. OR/1-2 | 1. TS=(Self care OR self-care) OR TS=(professional family relation*) OR TS=(telephone) OR TS=(professional patient relation*) OR TS=(attitude* of health personnel*) OR TS=(cellular phone) OR TS=(handheld computer) OR TS=(self-efficacy) OR TS=(self efficacy OR self-efficacy OR confidence) OR TS=(ADL) OR TS=(activit* of daily living) 2. TS=(self-help devices) OR TS=(community health service*) OR TS=(rehabilitation) OR TS=(responsib* OR autonomy*) OR TS=(manag* OR cope* OR coping) OR TS=(disease management) OR TS=(expert patient) OR TS=(educat* OR training OR skills* OR knowledge) 3. TS=(monitor* OR self-monitor* OR selfmonitor*) OR TS=(tele-health OR tele-medicine OR tele-care OR telehealth OR telemedicine OR telecare) OR TS=(short message service* OR SMS OR mobile phone* OR text message*) OR TS=(care plan OR action plan) OR TS=(personalised action plan) OR TS=(CBT) OR TS=(psychoeducation*) OR TS=(personal health budget*) OR TS=(financial AND control) 4. TS=(professional* OR clinician*) AND TS=(development) 5. TS=(patient* OR individual* OR person* OR client*) AND TS=(remind* OR feeback) 6. TS=(access* OR provi*) AND TS=(information OR record* OR result*) 7. TS=(home OR environment* OR living OR assistive) AND TS=(adaptation OR modif* OR equipment OR technolog*) 8. TS=(cognitive or psychological or interpersonal or relaxation or biofeedback) AND TS=(therap* or intervention* or program*) 9. TS=(peer or patient or emotional or social or psychosocial) AND TS=( support or group*) 10. TS=(financial or monetary or payment* or discount or service*) AND TS=( incentiv*) 11. OR/1-10 | 1. TI=(randomized controlled trial*) OR TS=(RCT) OR TS=(random allocation) OR TS=(double blind method) OR TS=(single blind method) OR TS= (clinical trial*) OR TS=(controlled clinical trial*) OR TS=(multicentre study OR multicenter study) OR TS=(clinical trial*) OR TI=(controlled clinical trial*) 2. TS=(phase i OR phase 1) OR TS=(phase ii OR phase 2) OR TS=(phase iii OR phase 3) OR TS=(phase iv OR phase 4) 3. TS=(control group*) OR TS=(usual care) OR TS=(randomly allocated) 4. TS=(quasi experimental) OR TS=(pseudo experimental) OR TS=(controlled before-and-after) OR TS=(interrupted time series design) 5. OR/1-4 |
| SCIENCE DIRECT | pub-date > 1989 and (health litera* OR medical litera* OR functional litera* OR health promotion OR health education OR patient education OR communication barrier* OR education* status OR attitude to health OR health knowledge, attitude*, practice OR comprehension OR litera*) | pub-date > 1989 and (asthma or bronchial asthma) | pub-date > 1989 and (self care OR self-care OR self management OR self-management OR selfmonitor* OR self-monitor* OR audiovisual aid* OR self-efficacy OR expert patient) | pub-date > 1989 and (randomized control trial* OR controlled clinical trial* OR quasi experimental OR pseudo experimental OR interrupted time series design*). |
| COCHRANE | 1. [Health Literacy] 2. [Reading] 3. [Comprehension] 4. [Health Promotion] 5. [Health Education] 6. [Patient Education as Topic] 7. [Communication] 8. [Communication Barriers] 9. [Health Knowledge, Attitudes, Practice] 10. [Comprehension] AND [Educational Status] 11. [Attitude to Health] 12. [Drug Labeling] AND [Comprehension] AND [Patients] 13. OR/1-12 | 1. [Asthma] 2. [Bronchial Spasm] 3. [Bronchoconstriction] 4. [Bronchial Hyperreactivity] 5. [Respiratory Hypersensitivity] 6. OR/1-5 | 1. [Self Care] 2. [Professional-Family Relations] 3. [Telephone] 4. [Professional-Patient Relations] 5. [Attitude of Health Personnel] 6. [Computers, Handheld] 7. [Self Efficacy] 8. [Activities of Daily Living] 9. [Self-Help Devices] 10. [Community Health Services] 11. [Rehabilitation] 12. [Self-Management] 13. [Personal Autonomy] AND [Patients] 14. [Adaptation, Psychological] 15. [Disease Management] 16. [Formative Feedback] 17. [Reminder Systems] AND [Patients] 18. [Staff Development] 19. [Telemedicine] 20. [Text Messaging] 21. [Cognition] 22. [Cognitive Therapy] 23. [Self-Help Groups] 24. OR/1-23 | 1. [Randomized Controlled Trial] 2. [Control Groups] 3. [Random Allocation] 4. [Double-Blind Method] 5. [Single-Blind Method] 6. [Multicenter Study] 7. [Controlled clinical trials, randomized] 8. [Quasi experimental studies] 9. [Controlled before-after studies] 10. [interrupted time series analysis] 11. OR/1-10 |
